# Profiling the Stromal and Vascular Heterogeneity in Patient-derived Xenograft Models of Head and Neck Cancer: Impact on Therapeutic Response

**DOI:** 10.3390/cancers11070951

**Published:** 2019-07-06

**Authors:** Margaret Folaron, Mihai Merzianu, Umamaheswar Duvvuri, Robert L. Ferris, Mukund Seshadri

**Affiliations:** 1 Department of Oral Oncology, Roswell Park Comprehensive Cancer Center, Buffalo, NY 14263, USA; 2 Department of Pathology and Laboratory Medicine, Roswell Park Comprehensive Cancer Center, Buffalo, NY 14263, USA; 3 Department of Otolaryngology, University of Pittsburgh—Hillman Cancer Center, Pittsburgh, PA 15261, USA; 4 Department of Dentistry and Maxillofacial Prosthetics, Roswell Park Comprehensive Cancer Center, Buffalo, NY 14263, USA

**Keywords:** patient derived xenografts, HNSCC, angiogenesis, heterogeneity, VDAs, MRI

## Abstract

Head and neck squamous cell carcinomas (HNSCC) represent a group of epithelial neoplasms that exhibit considerable heterogeneity in clinical behavior. Here, we examined the stromal and vascular heterogeneity in a panel of patient-derived xenograft (PDX) models of HNSCC and the impact on therapeutic response. Tumor sections from established tumors were stained for p16 (surrogate for human papillomavirus (HPV) infection), stromal (Masson’s trichrome) and vascular (CD31) markers. All PDX models retained the HPV/p16 status of the original patient tumor. Immunohistochemical evaluation revealed the presence of multiple vessel phenotypes (tumor, stromal or mixed) in the PDX panel. Vascular phenotypes identified in the PDX models were validated in a tissue microarray of human HNSCC. Treatment with a microtubule targeted vascular disrupting agent (VDA) resulted in a heterogeneous antivascular and antitumor response in PDX models. The PDX with the tumor vessel phenotype that exhibited higher CD31+ vessel counts and leaky vasculature on magnetic resonance imaging (MRI) was sensitive to VDA treatment while the PDX with the stromal vessel phenotype was resistant to therapy. Collectively, our results demonstrate the phenotypic and functional vascular heterogeneity in HNSCC and highlight the impact of this heterogeneity on response to antivascular therapy in PDX models of HNSCC.

## 1. Introduction

Head and neck squamous cell carcinomas (HNSCC) are aggressive epithelial neoplasms that can arise in several sites (e.g., oral cavity, pharynx, larynx) within the upper aero-digestive tract and exhibit considerable heterogeneity in clinical behavior [1,2]. Multiple risk factors including tobacco, alcohol and human papillomavirus (HPV) infection have been implicated in the pathogenesis of HNSCC [3,4]. Although patients with early stage disease exhibit a favorable response to therapy, a majority of HNSCC patients present with advanced stage disease which is associated with a poor prognosis [2,5]. As such there is a critical need to investigate novel treatment strategies for patients that suffer from these esthetically and functionally debilitating cancers.

In this regard, patient-derived xenograft (PDX) models of cancer provide an important platform for understanding disease biology and examining therapeutic activity of novel agents [6,7,8]. Studies by us and others have previously reported on the ability of PDX models of HNSCC to reliably mimic the histopathology and molecular characteristics of human disease [9,10,11]. However, systematic examination of the vascular heterogeneity in PDX models of HNSCC has not been performed. To address this gap in knowledge, in the present study, we established and characterized the vascular phenotypes in a panel of PDX models of HNSCC. Vascular phenotypes identified in the PDX models were validated in a tissue microarray (TMA) of human HNSCC. The therapeutic impact of this angiogenic heterogeneity was evaluated by examining tumor response to a microtubule targeted vascular-disrupting agent (VDA), EPC2407 (Crolibulin™). The agent competitively binds to the colchicine binding site on microtubules and has demonstrated preclinical anticancer activity in breast, lung, prostate and brain tumor models [12,13,14,15]. However, the antivascular and antitumor activity of the agent has not been evaluated in PDX models of HNSCC.

## 2. Results

### 2.1. Establishing a Panel of PDX Models of HNSCC

**Figure 1:**
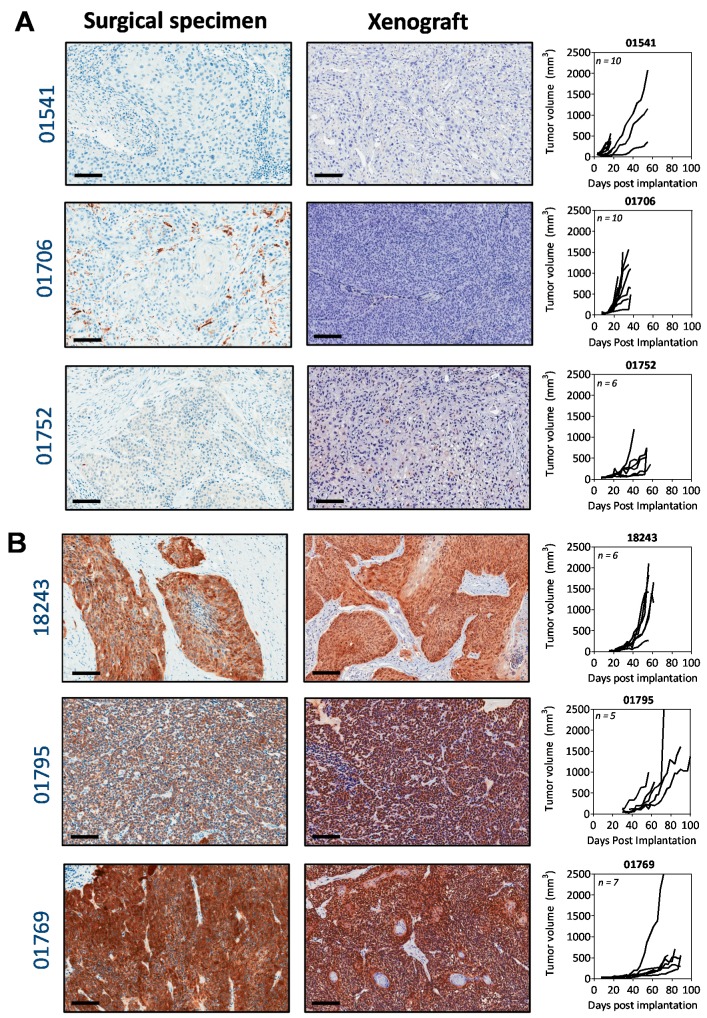
Patient-derived xenografts retain the human papillomavirus (HPV) status of the donor tumor. Panel of photomicrographs of the original patient sample (surgical specimen) and corresponding xenograft stained for p16 as a marker of HPV status (*n* = 3–4 samples per patient-derived xenograft (PDX)). Images are shown for three p16-negative samples (**A**) and three p16-positive samples (**B**). Images were captured at 20× magnification, the scale bar represents 100 μm. Individual tumor volume curves of the three p16− PDX and the three p16+ PDX are shown on the right (*n* = 5–10 tumors per PDX type).

We first established a panel of HPV-positive and HPV-negative PDX models of HNSCC. Information on the patient characteristics and donor tumors is summarized in the Supplementary Materials Table S1. Staining of tumors for p16 expression was utilized as a surrogate marker of HPV infection [16,17,18]. We performed p16 immunostaining on the donor tumor tissue (surgical specimen), and corresponding established PDX of all six models to determine their HPV status. All PDX models retained the HPV/p16 status of the original patient tumor (Figure 1). The three p16+ surgical specimens and corresponding PDX showed strong nuclear and cytoplasmic staining of p16 (Figure 1B). Tumor growth kinetics of successfully established subcutaneous xenografts were evaluated by calculating the tumor volume from caliper measurements over 100 days (*n* = 5–14 tumors per PDX model). The individual growth curves for the six PDX models are also shown. Pooled tumor doubling times from all six models calculated from these individual growth curves revealed shorter times (*p* = 0.01) for p16− PDX (7.8 ± 0.7 days) compared to the p16+ PDX (11 ± 1 days). Detection of HPV16 E6 DNA by PCR of the primary tumor tissue and the matching xenograft was used as a validation measure (Supplementary Materials Figure S1). Good concordance was observed between the p16 and HPV status (Supplementary Materials Table S2). The three p16 positive (p16+) models were also HPV positive (HPV+) and the three p16 negative (p16−) models were also HPV negative (HPV−).

### 2.2. Profiling the Stromal and Vascular Heterogeneity in PDX Models of HNSCC.

**Figure 2:**
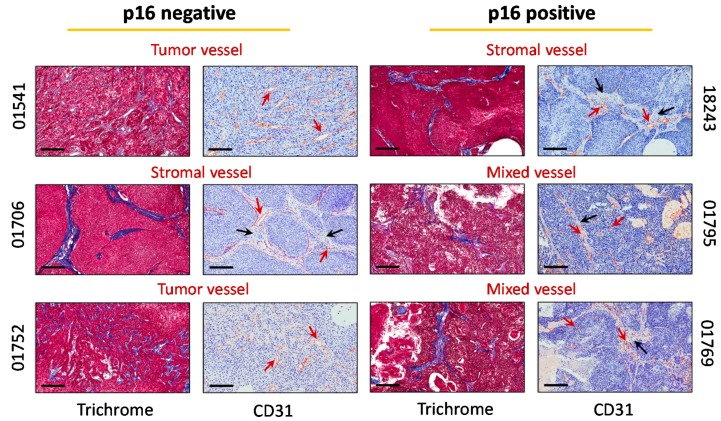
Stromal and vascular heterogeneity in PDX models of head and neck squamous cell carcinomas (HNSCC). Panel of images represent photomicrographs of Masson’s trichrome and CD31 stained tumor sections of the three p16-negative and the three p16+ PDX models of HNSCC. All images were captured at 20× magnification. Tumors were classified as tumor vessel (TV), stromal vessel (SV) or mixed phenotype based on the location and distribution of CD31+ vessels (*n* = 4–5 fields per tumor section, *n* = 3–5 samples per xenograft type). Black arrows point to stroma and red arrows indicate CD31+ blood vessels. Images are shown at 20× magnification, scale bar represents 100 µm.

Next, we performed immunohistochemical and histologic evaluation of established tumors to characterize the tumor architecture of our six PDX models. Tumor sections were stained for Masson’s trichrome (Figure 2, Trichrome) showed regions of reactive stroma evidenced by blue bands (01706 and 18243). The two p16+ PDX (01795, 01769) showed lower intensity of trichrome positivity (Figure 2). CD31 immunostaining of tumor sections was performed to profile the vascular heterogeneity of the six PDX models (Figure 2, CD31). The location and distribution of CD31+ vessels were evaluated to define the vessel phenotype of each individual PDX model. Consistent with published observations in lung cancer [19], CD31-immunostained tumor sections revealed the presence of three distinct vascular phenotypes across the six PDX models: A tumor vessel (TV) phenotype in which blood vessels were distributed throughout the tumor (01541, 01752), a stromal vessel (SV) phenotype in which a majority of the vessels (red arrows) were restricted to the infiltrating stroma (black arrows) surrounding the tumor cells (01706, 18243), and a mixed vessel phenotype that showed CD31+ vessels in the stroma and between islands of tumor cells (01795, 01769).

### 2.3. Validation of Vascular Phenotypes in Human HNSCC

**Figure 3:**
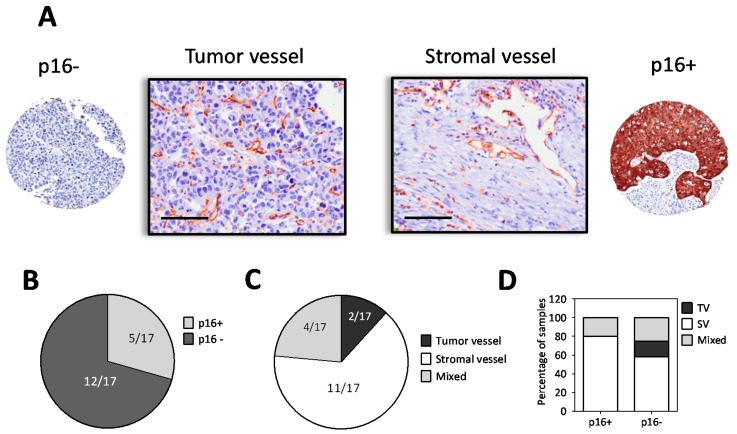
Heterogeneity in vascular phenotype of p16+ and p16− human HNSCC. (**A**) Photomicrographs of p16 and CD31-immunostained tissue microarray (TMA) cores of human HNSCC. Images are shown at 20× magnification, scale bar represents 100 µm. A p16 negative (p16−) sample with a tumor vessel (TV) phenotype and a p16 positive (p16+) sample with a stromal vessel (SV) phenotype is shown. Vessels were identified as CD31+ structures with a visible lumen. (**B**) Incidence of p16+ and p16− samples in the TMA. (**C**) Incidence of the three different vessel phenotypes in the TMA. (**D**) Distribution of the vessel phenotypes in the TMA stratified by p16 status.

To validate our preclinical observations, we utilized a tissue microarray (TMA) analysis using surgical samples of human HNSCC (*n* = 17; Supplementary Materials Table S3). Immunostaining of the TMA for p16 and CD31 was performed to determine the association between HPV status and tumor vascular phenotype (Figure 3). Consistent with our observations in the PDX models, we observed existence of all three vessel phenotypes in human HNSCC. Figure 3A shows photomicrographs of a p16− and p16+ tumor along with the corresponding CD31 stained image (20× magnification). A majority of the samples in the TMA (12/17; 70%) were negative for the HPV marker p16 (Figure 3B). Eleven out of 17 samples (65%) exhibited the SV phenotype while 2/17 samples (~12%) exhibited the TV phenotype (Figure 3C). The remaining samples (23%) showed a mixed phenotype with vessels present in the stroma and within islands of tumor cells. When stratified by p16 status, 80% of p16 positive (p16+) tumors exhibited the SV phenotype, while the remainder showed a mixed vessel phenotype. In comparison, a heterogeneous distribution of vascular phenotypes was observed in p16− tumors: 60% of the p16− samples exhibited the SV phenotype, ~25% showed a mixed vessel phenotype and ~15% showed TV phenotype (Figure 3D).

### 2.4. Early Pathologic Response and Therapeutic Efficacy of VDA Therapy in PDX Models of HNSCC

**Figure 4:**
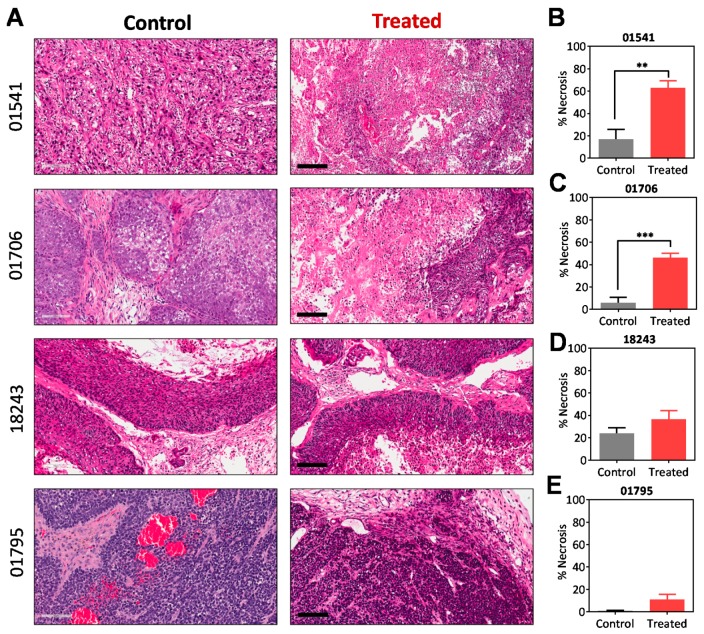
Pathologic response of p16− PDX and p16+ PDX to EPC2407. (**A**) Hematoxylin and eosin (H&E) stained tumor sections from control and vascular disrupting agent (VDA) treated p16− PDX (01541, 01706) and p16+ PDX (18243, 01795). Images are shown at 20× magnification, scale bar represents 100 µm. Corresponding bar graphs of tumor necrosis (%) for control and treated tumors (*n* = 4–5 tumors per PDX; *n* = 3–4 fields per tumor) for all 4 PDX models are shown on the right (**B**–**E**). ** *p* < 0.01; *** *p* < 0.001.

Next, we evaluated the response of four PDX models with varying stromal and vascular phenotype to the microtubule-targeted VDA, EPC2407 (Crolibulin™). Given the cardiovascular safety concerns with VDAs [20], we performed echocardiography in mice to assess changes in cardiac function before and after VDA treatment (Supplementary Materials Figure S2). Mice were administered EPC2407 (20 mg/kg; intravenously (i.v.), twice a week for two weeks) and cardiovascular parameters were measured 24 h after each dose. Treatment with EPC2407 did not result in any significant changes in cardiac output, fractional shortening, or ejection fraction at this dose. We therefore examined the antitumor activity of EPC2407 using this safe dose and schedule. Pathologic response of tumors to EPC2407 was evaluated by quantification of tumor necrosis on hematoxylin and eosin (H&E) sections of control- and treated-tumors in the four PDX models (Figure 4A). Quantification of tumor necrosis (relative to whole tumor area) showed a significant increase following VDA treatment compared to control tumors in the two p16-negative PDX models (Figure 4B, *p* < 0.01 for 01541; Figure 4C, *p* < 0.001 for 01706). In comparison, differences in tumor necrosis between control and VDA-treated tumors in the two p16+ models were not statistically significant (Figure 4D,E).

**Figure 5:**
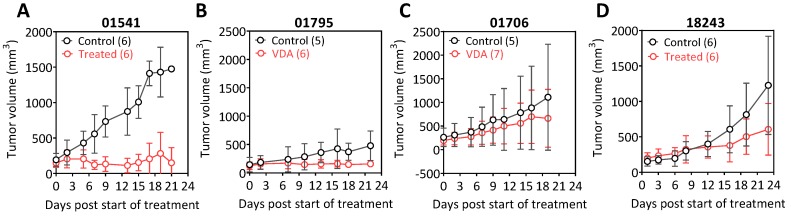
Response of p16+ and p16− PDX models of HNSCC to vascular-targeted therapy. Temporal changes in tumor volumes in p16-negative 01541 (**A**), 01706 (**B**) and p16-positive PDX, 18243 (**C**), 01795 (**D**) models of HNSCC following treatment with the microtubule targeted vascular disrupting agent (VDA) EPC2407. Individual plots represent response of control (black circles) and EPC-treated tumors (red circles) for the four PDX models are shown. The p16− PDX (01541) with the tumor vessel phenotype was the most responsive to vascular targeted therapy.

Analysis of long-term response at this dose showed marked heterogeneity in tumor growth inhibition following VDA therapy across the PDX models. Individual growth curves of control (black circles) and EPC-treated tumors (red circles) from two p16− PDX, 01541 (A), 01706 (B) and two p16+ PDX, 01795 (C), 18243 (D) are shown in Figure 5. The p16− PDX 01541 with the TV phenotype was the most responsive to vascular targeted therapy and exhibited marked tumor growth inhibition over three weeks post treatment (Figure 5A). The acute pathologic response to EPC2407 observed in the p16− PDX 01706 did not translate into durable tumor growth inhibition (Figure 5B). The p16+ PDX 01795 with the mixed vessel phenotype also showed evidence of tumor growth inhibition although the magnitude of the response was less pronounced compared to the p16− PDX 01541 (Figure 5C). The p16+ PDX 18243) with the SV phenotype also showed minimal tumor growth inhibition following treatment (Figure 5D).

### 2.5. Impact of Phenotype on the Susceptibility of Vessels to VDA Therapy

**Figure 6:**
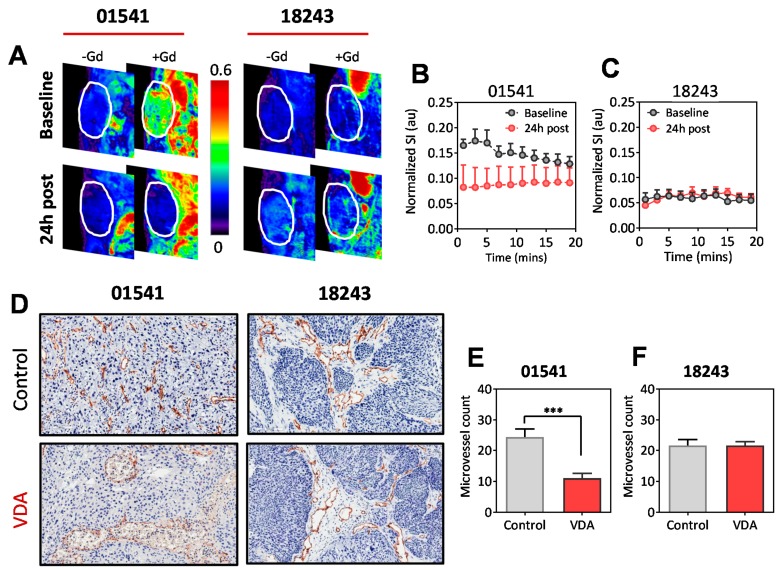
Functional imaging of heterogeneity in susceptibility to vascular-targeted therapy in vivo. (**A**) Normalized T1-weighted images of the p16− PDX 01541 and p16+ PDX 18243 at baseline and 24 h post treatment with the microtubule targeted VDA, EPC2407. Pseudo-colorized images of the PDX before and after contrast (Gd) are shown to visualize contrast enhancement within the tumor. Dynamic changes in T1-weighted signal intensity values (as a measure of contrast enhancement) of the p16− PDX (01541; **B**) and p16+ PDX (18243; **C**) at baseline (gray circles) and 24 h post EPC2407 treatment (red circles). (**D**) CD31-immunostained tumor sections confirmed the differential vascular response between the PDX models with marked vascular damage seen in the 01541 model (bottom; VDA). In comparison, EPC-treated tumors in the 18243 model appeared similar to controls with intact CD31+ vessels visible in the tumor stroma. A significant reduction in microvessel count was seen in the 01541 PDX (**E**) following EPC treatment (red bar; VDA). EPC-treated tumors showed comparable vessel counts to control tumors in the 18243 PDX (**F**). *** *p* < 0.001.

Finally, to gain some mechanistic insight into the differential response to EPC2407 observed in our PDX models, we examined the impact of tumor phenotype on the susceptibility of vessels to VDA therapy. To this end, acute changes in vascular function following EPC2407 treatment were studied in the sensitive PDX (p16-01541; tumor vessel) and the resistant PDX (18243; stromal vessel) using dynamic contrast-enhanced magnetic resonance imaging (DCE-MRI). As shown in Figure 6A, DCE-MRI revealed a higher leakage of the magnetic resonance contrast agent, gadolinium (+Gd) in the p16− PDX 01,541 at baseline (prior to treatment) compared to the p16+ PDX 18243. Twenty four hours following administration of EPC2407 (20 mg/kg, i.v.), a significant reduction in contrast enhancement (Figure 6A,B) was seen in the 01,541 PDX reflective of tumor vascular damage following treatment. Corresponding CD31 immunostaining showed a marked reduction in vessel number (Figure 6D,E) following treatment. In comparison, the PDX 18,243 showed no change in enhancement characteristics on DCE-MRI (Figure 6A,C). Immunohistochemical analysis did not reveal any change in vessel count or tumor necrosis between control and treated tumors in the 18,243 PDX model (Figure 6D,F). These observations suggest tumor vascular phenotype contributes to the differential susceptibility of tumors to microtubule-targeted VDA therapy in PDX models of HNSCC.

## 3. Discussion

Head and neck cancers exhibit considerable heterogeneity in biologic behavior and therapeutic response in humans. It would therefore be important to develop and characterize preclinical models that can adequately capture this heterogeneity of these aggressive cancers. In the present study, we demonstrated the successful establishment and characterization of a panel of HPV− and HPV+ PDX models of HNSCC with varying vascular phenotypes. Our results illustrate the degree of vascular heterogeneity in PDX models of HNSCC and its impact on therapeutic response.

To date, a few groups have established HPV+ and HPV− PDX models of HNSCC [10,11]. The HPV oncoprotein E7 results in degradation of the Rb family of proteins, which leads to activation of E2F, and uncontrolled transcription of S phase genes. This in turn results in expression of p16INK4a, an inhibitor of cyclin-dependent kinases in a negative feedback loop [16,17]. We therefore performed p16 staining on the original donor tumor tissue and the matching PDX. Similar to these published reports, we observed good concordance between p16/HPV status of original tumor and established PDX. Studies performed by us and others have previously reported on the angiogenic heterogeneity and its impact on drug delivery and therapeutic response using PDX models of HNSCC [9,21,22]. Smith et al. have documented the presence of multiple vascular phenotypes (stromal, tumor or mixed) in lung and head and neck cancers [19]. However, to the best of our knowledge, vascular phenotyping of HPV+ and HPV− HNSCC has not been previously reported. In the present study, we established a panel of p16+ and p16− PDX models of HNSCC and examined the influence of HPV on vascular phenotype. Our examination showed the presence of, S.V.; TV and mixed phenotypes in our PDX panel. We validated our observations in the PDX models using a limited tissue microarray (TMA) of human HNSCC samples. Immunostaining of the TMA for p16 and CD31 was performed to determine the association between HPV status and tumor vascular phenotype. Similar to the PDX models, we observed all three vessel phenotypes in the TMA. Our TMA analysis revealed a higher incidence of stromal vessel phenotype in p16+ tumors compared to p16− tumors, which exhibited a considerable heterogeneity in vessel phenotype (SV, TV and mixed). While we did not investigate the molecular differences between the different vascular phenotypes, our findings are consistent with a report by Baruah et al., in which strong VEGF expression was seen in tumor and stromal cells in p16-negative tumors, while VEGF expression in p16+ tumors was restricted primarily to the stroma [23]. Troy et al. have also reported differences in NOTCH1 and VEGF expression between HPV+ and HPV− HNSCC samples [24]. Similarly, Hauff et al., have shown higher stromal collagen, decreased vimentin and matrix metalloproteinase levels in HPV+ head and neck tumors compared to HPV− HNSCC [25]. These published observations together with the findings of the present study, add to the limited body of evidence on the influence of HPV on angiogenic heterogeneity in HNSCC.

We examined the therapeutic impact of this vascular heterogeneity in our PDX models. Several clinical studies have demonstrated that patients with HPV positive tumors respond more favorably to conventional anticancer therapies (surgery, chemotherapy and radiation) than HPV− tumors [18,26,27]. However, the response of HPV+ and HPV− tumors to vascular targeted therapy has not been systematically examined. In this study, using PDX models, we examined the impact of HPV on tumor angiogenesis and response of HPV+ and HPV− HNSCC to a microtubule-targeted tumor-VDA, EPC2407 (Crolibulin™) that has demonstrated potent antivascular and antitumor activity in experimental models of breast, prostate and brain tumors [12,13,14,15]. The agent has also undergone early phase clinical evaluation in cancer patients [clinicaltrials.gov NCT01240590]. We utilized DCE-MRI, a non-invasive functional imaging method [9] to examine the early vascular response of PDX to VDA treatment. DCE-MRI revealed greater contrast agent leakage in the HPV− PDX at baseline compared to the HPV+ PDX. Consistent with previous observations in melanomas [28] and lung cancers [19], the PDX 01541 with the tumor vessel phenotype that exhibited higher CD31+ vessel counts and leaky vasculature on MRI was sensitive to EPC2407 treatment while HPV+ 18243 tumors with the stromal vessel phenotype was resistant to therapy. Long-term monitoring of therapeutic response revealed marked tumor growth inhibition that conferred a survival benefit in the HPV− PDX 01541 with no therapeutic benefit seen in the HPV+ 18243 PDX. Our results highlight differential sensitivity of HPV+ and HPV− PDX to vascular-targeted therapy in vivo. Although further investigation in additional HPV+ and HPV− models is needed, our observation is consistent with the results of the Danish trial examining the combination of radiation therapy with the hypoxic sensitizer, Nimarazole. Retrospective assessment of pretreatment p16 expression suggested that hypoxic modification improved outcomes in HPV/p16− tumors but did not provide any significant benefits in HPV/p16+ tumors [29]. Similarly, in a Phase II study conducted in Her2-negative breast cancers patients, clinical response to bevacizumab was primarily seen in patients with high baseline tumor microvessel density [30]. Studies have also shown that tumors with p53 mutations facilitate a pro-angiogenic, hyper-proliferative phenotype and a higher rate of clinical benefit with VEGF/R inhibitors [31,32]. As such, the role of antiangiogenic agents and VDAs in the treatment paradigm for HNSCC is unclear. These observations along with our results suggest that p16− head and neck tumors that often harbor p53 mutations and exhibit increased angiogenesis could be more susceptible to vascular-targeted therapies such as VDAs and VEGF inhibitors. This is particularly relevant since patients with HPV+ HNSCC show improved outcomes with existing treatment options while the therapeutic needs of ‘traditional’ HPV− HNSCC remain unmet.

Several caveats to clinical translation of our preclinical study findings warrant careful consideration. First, given the need to establish tumors in immunodeficient hosts, the cross-talk between immune cells, stromal architecture and vascular phenotype cannot be studied in PDX models. Second, our studies were conducted using PDX models established beneath the skin (ectopic). It would be important to conduct studies using orthotopic models to validate our observations. Finally, we examined the sensitivity of p16+ and p16− PDX to VDA monotherapy. However, it would be important to study the response of a panel of PDX models to VDAs in combination with clinically utilized chemotherapeutic regimens. It is important to note that in established PDX, the tumor cells are of human origin while the stroma and the vessels are of murine origin. Hence, the observed activity of antivascular agents may not reflect the true sensitivity of human blood vessels to such agents. Nevertheless, to the best of our knowledge, this is the first report on heterogeneity in tumor vascular architecture, perfusion, oxygenation and response to vascular targeted therapy in PDX models of HNSCC.

## 4. Materials and Methods

### 4.1. Animals

Experimental studies were carried out using eight-to-twelve week old female C.B 17 severe combined immunodeficient (SCID) mice (C.B-Igh-1^b^/IcrTac-Prkd^scid^; Laboratory Animal Shared Resource, RPCI) with an average body weight ~20 g. Mice were kept in sterile micro-isolator cages (4–5 mice per cage) in a pathogen-free environment and provided with standard chow/water and maintained on 12 h light/dark cycles in a high efficiency particulate air (HEPA)-filtered environment. Experimental procedures were performed under aseptic conditions and in accordance with protocols approved by the Institutional Animal Care and Use Committee (1183M, Animal welfare assurance number A-3143-01).

### 4.2. Procurement of Human Tumor Tissue and Establishing of Xenografts

A head and neck pathologist (M.M) analyzed the tumors after resection and selected viable non-diagnostic tumor tissue for transplantation into mice. Procurement of tumor tissues was performed under an institutional review board (IRB) approved Non-Human Subjects Research Protocol NHR024912 (Original approval date: 05/10/2012) at Roswell Park Comprehensive Cancer Center. We have previously described the workflow for establishing PDX models of HNSCC [9]. Briefly, procured tumor tissue was transferred to the laboratory in culture media for transplantation into SCID mice. Tumors were implanted subcutaneously in the belly during the initial phase (engraftment phase) and upon successful establishment, subsequently passaged into recipient mice in the flank (expansion phase) for experimental studies using aseptic techniques. All experimental studies were conducted in PDX that were passaged four to five times.

### 4.3. Immunostaining of PDX

Mice were humanely euthanized according to recommendations of the Panel on Euthanasia of the American Veterinary Medical Association and tumor tissues resected for further processing. Tumor tissues were fixed in 10% neutral buffered formalin (Sigma-Aldrich, St. Louis, MA, USA) or zinc fixative (BD Biosciences, San Diego, CA, USA) and embedded in paraffin. Tissues were sectioned at a thickness of 4 μm, mounted on positively charged slides, and stained for p16 (Roche Clone E6H4; Catalog #9517, Indianapolis, IN, USA), CD31 (BD Pharmingen, Catalog #550274, San Jose, CA, USA), and Masson’s Trichrome (Poly Scientific R&D Corporation; Catalog #K037, Bay Shore, NY, USA). Whole tumor sections were captured and digitized using the ScanScope XT system (Aperio Technologies, Vista, CA, USA). Images were captured at 20× magnification (3–5 tumors per PDX model; 4–5 fields per tumor). Assessment of p16 status and location of CD31-stained vessels was performed under the supervision of a board-certified head and neck pathologist (M.M.). p16 was considered positive if tumors exhibited strong, diffuse cytoplasmic and nuclear labeling of ≥75% neoplastic cells.

### 4.4. Image Segmentation and Morphometric Analysis

Processed whole tumor sections were analyzed using Analyze software (AnalyzeDirect, version 7.0; Overland Park, KS, USA) and ImageJ (National Institutes of Health, Bethesda, MD, USA). Image segmentation and morphologic analysis of CD31 and Masson’s trichrome stained tumor sections was performed to determine location (vascular phenotype) and number (vessel density). Image segmentation was performed through separation of color channels using the color deconvolution plug-in (Image J, National Institutes of Health). CD31 and trichrome stained sections were captured for the same region of the tumor. A region of interest (ROI) was created for each vessel in CD31 stained photomicrographs to calculate microvessel count in a given field. Vascular phenotype was determined based on the predominant (60% or greater) location of blood vessels, surrounding tumor cells (tumor vessel) or in the stroma (stromal vessel). Tumors exhibiting a comparable distribution of vessels in the stroma and around tumor cells were classified as “mixed vessel” phenotypes based on a previous study by Smith et al. [19]. Three to five tumors per PDX model were sectioned, stained and evaluated.

### 4.5. Human HNSCC Tissue Microarray

Banked human tumor tissue samples (*n* = 17) were used to construct a tissue microarray (TMA) through the Pathology Resource Network at Roswell Park. Information on the patient characteristics and site of origin for the samples in the TMA are summarized in Supplementary Materials Table S3. Three to four cores were taken from each patient sample. The cores were stained with commercially available antibodies: p16 (Roche Clone E6H4; Catalog #9517, Indianapolis, IN, USA) and CD31 (Dako; Catalog #JC70A; Agilent Technologies, Santa Clara, CA, USA). Paraffin sections were cut at 4μm, placed on charged slides and stained. Images were digitized using the ScanScope XT system and ImageScope software (Aperio Technologies, Vista, CA, USA). One field per tumor core (10× magnification) was captured and analyzed using Analyze software (AnalyzeDirect, version 7.0; Overland Park, KS, USA) to determine the vessel phenotype.

### 4.6. Drug Treatment

EPC2407 (kindly provided by EpiCept Corporation, Tarrytown, NY, USA) was dissolved in vehicle comprised of phosphate-buffered saline, lutrol and polyethylene glycol at a concentration of 5 mg/mL and administered intravenously (i.v.) at a dose of 20 mg/kg administered i.v. two times per week for three weeks.

### 4.7. Therapeutic Response Assessment

Caliper measurements were taken thrice weekly once a palpable tumor was apparent and used to estimate tumor volume. Tumor volume was calculated using the formula V = (*l* × *w*^2^)/2 where *l* represents the longest axis of the tumor and *w* represents the axis perpendicular to the long axis. Calculated values were reported as the mean ± standard deviation of the mean. Tumor growth kinetics of successfully established subcutaneous xenografts were evaluated by calculating the tumor volume from caliper measurements over 100 days (*n* = 5–12 tumors per PDX)

### 4.8. Magnetic Resonance Imaging

Experimental MRI examinations were performed using a 4.7T/33-cm horizontal bore magnet (GE NMR Instruments, Fremont, CA, USA) incorporating AVANCE digital electronics (Bruker Biospec with Paravision 3.0.2; Bruker Medical Inc., Billerica, MA, USA). Animals were anesthetized using 2.5% Isoflurane (Benson Medical Industries, Markham, O.N.; Canada) prior to and during imaging. Three-dimensional spoiled gradient echo scans [TR = 50 ms, TE = 3.5 ms, FOV 4.8 × 3.2 × 3.2 cm] were acquired before (three pre contrast) and after (10 post contrast) intravenous injection of the MR contrast agent, Gadofosveset trisodium (Ablavar™; Lantheus Medical Imaging, N. Billerica, MA, USA) at a dose of 0.1 mmol/kg. Following image acquisition, raw image sets were transferred to a processing workstation and converted into Analyze™ format (AnalyzeDirect, version 10.0; Overland Park, KS, USA). Tumor enhancement maps were calculated from normalized signal intensity values (tumor/phantom) as previously described [33].

### 4.9. Sample Sizes and Statistics

All statistical analysis was performed using GraphPad version 7.00 for Windows (GraphPad Software, San Diego, CA, USA). Non-invasive imaging was performed using 3–5 tumor bearing mice per PDX model. Tumors were excised for correlative immunohistochemistry and histology (*n* = 3–5 tumors per PDX model). Comparisons of individual values between different cohorts were analyzed using an unpaired two-tailed student’s t test. *p*-values < 0.05 were considered statistically significant.

## 5. Conclusions

The results of our present study demonstrate the considerable degree of phenotypic and functional vascular heterogeneity in PDX models of HNSCC. Our observations also highlight the utility of non-invasive functional imaging methods such as DCE-MRI in profiling the heterogeneity in vascularity and response to antivascular therapy in PDX models. Integration of imaging data on tumor vascular phenotypes with underlying histopathologic and molecular profiles could prove valuable in identifying tumors (patients) that might benefit from vascular-targeted therapy.

## Supplementary Materials

The supplementary materials are available online at https://www.mdpi.com/2072-6694/11/7/951/s1. Figure S1: PDX models of HNSCC retain the HPV/p16 status of the original patient tumor. PCR based detection of HPV 16 E6 region in HNSCC surgical specimens and matching six PDX models evaluated in the study. The HPV status of the donor human tumor tissue was retained in the PDX models. In agreement with p16 immunohistochemistry, the three p16+ PDX and their original patient tumors exhibited bands for p16E6, Figure S2: Effect of EPC2407 treatment on cardiovascular function in mice. (A) Long axis b-mode ultrasound images of a control (left) and treated (right) mouse heart at baseline (top), 24 h following one dose of EPC2407 (middle; 24 h post 1st), and 24 h following four doses of EPC2407 (bottom; 24 h post 4th). (B–D) Corresponding bar graphs of cardiac output (left), fractional shortening (middle), and ejection fraction (right) (n = 5 mice/cohort) are shown in the bottom, Table S1: Patient characteristics of the PDX models generated in the study, Table S1: Comparative assessment of histology and p16 status of surgical donor tumor tissue and corresponding patient-derived xenografts. We did not observe any relationship between tumor differentiation, vascular phenotype and growth rate. (Well diff—well differentiated SCC; Poorly diff—poorly differentiated SCC; Mod. diff—Moderately differentiated SCC), Table S3: Patient characteristics of the tissue microarray.

## References

[1] Patel S.G., Shah J.P. (2005). TNM staging of cancers of the head and neck: Striving for uniformity among diversity. CA Cancer J. Clin..

[2] Mroz E.A., Tward A.D., Pickering C.R., Myers J.N., Ferris R.L., Rocco J.W. (2013). High intratumor genetic heterogeneity is related to worse outcome in patients with head and neck squamous cell carcinoma. Cancer.

[3] Park B.J., Chiosea S.I., Grandis J.R. (2010). Molecular changes in the multistage pathogenesis of head and neck cancer. Cancer Biomark..

[4] Gillison M.L., Koch W.M., Capone R.B., Spafford M., Westra W.H., Wu L., Zahurak M.L., Daniel R.W., Viglione M., Symer D.E. (2000). Evidence for a causal association between human papillomavirus and a subset of head and neck cancers. J. Natl. Cancer Inst..

[5] Cognetti D.M., Weber R.S., Lai S.Y. (2008). Head and neck cancer: An evolving treatment paradigm. Cancer.

[6] Aparicio S., Hidalgo M., Kung A.L. (2015). Examining the utility of patient-derived xenograft mouse models. Nat. Rev. Cancer.

[7] Gengenbacher N., Singhal M., Augustin H.G. (2017). Preclinical mouse solid tumour models: Status quo, challenges and perspectives. Nat. Rev. Cancer.

[8] Byrne A.T., Alférez D.G., Amant F., Annibali D., Arribas J., Biankin A.V., Bruna A., Budinská E., Caldas C., Chang D.K. (2017). Interrogating open issues in cancer precision medicine with patient-derived xenografts. Nat. Rev. Cancer.

[9] Seshadri M., Merzianu M., Tang H., Rigual N.R., Sullivan M., Loree T.R., Popat S.R., Repasky E.A., Hylander B.L. (2009). Establishment and characterization of patient tumor-derived head and neck squamous cell carcinoma xenografts. Cancer Biol. Ther..

[10] Kimple R.J., Harari P.M., Torres A.D., Yang R.Z., Soriano B.J., Yu M., Armstrong E.A., Blitzer G.C., Smith M.A., Lorenz L.D. (2013). Development and characterization of HPV-positive and HPV-negative head and neck squamous cell carcinoma tumor grafts. Clin. Cancer Res..

[11] Peng S., Creighton C.J., Zhang Y., Sen B., Mazumdar T., Myers J.N., Lai S.Y., Woolfson A., Lorenzi M.V., Bell D. (2013). Tumor grafts derived from patients with head and neck squamous carcinoma authentically maintain the molecular and histologic characteristics of human cancers. J. Transl. Med..

[12] Gourdeau H., Leblond L., Hamelin B., Desputeau C., Dong K., Kianicka I., Custeau D., Boudreau C., Geerts L., Cai S.X. (2004). Antivascular and antitumor evaluation of 2-amino-4-(3-bromo-4,5-dimethoxyphenyl)-3-cyano-4H-chromenes, a novel series of anticancer agents. Mol. Cancer Ther..

[13] Cai S.X., Drewe J., Kemnitzer W. (2009). Discovery of 4-aryl-4H-chromenes as potent apoptosis inducers using a cell- and caspase-based Anti-cancer Screening Apoptosis Program (ASAP): SAR studies and the identification of novel vascular disrupting agents. Anticancer Agents Med. Chem..

[14] Kalmuk J., Folaron M., Buchinger J., Pili R., Seshadri M. (2015). Multimodal imaging guided preclinical trials of vascular targeting in prostate cancer. Oncotarget.

[15] Folaron M., Seshadri M. (2016). Bioluminescence and MR Imaging of the Safety and Efficacy of Vascular Disruption in Gliomas. Mol. Imaging Biol..

[16] Munger K., Baldwin A., Edwards K.M., Hayakawa H., Nguyen C.L., Owens M., Grace M., Huh K. (2004). Mechanisms of human papillomavirus-induced oncogenesis. J. Virol..

[17] Zheng Z., Baker C.C. (2006). Papillomavirus genome structure, expression dna post-transcriptional regulation. Front. Biosci..

[18] Ang K.K., Harris J., Wheeler R., Weber R., Rosenthal D.I., Nguyen-Tân P.F., Westra W.H., Chung C.H., Jordan R.C., Lu C. (2010). Human papillomavirus and survival of patients with oropharyngeal cancer. N. Engl. J. Med..

[19] Smith N.R., Baker D., Farren M., Pommier A., Swann R., Wang X., Mistry S., McDaid K., Kendrew J., Womack C. (2013). Tumor stromal architecture can define the intrinsic tumor response to VEGF-targeted therapy. Clin. Cancer Res..

[20] Subbiah I.M., Lenihan D.J., Tsimberidou A.M. (2011). Cardiovascular toxicity profiles of vascular-disrupting agents. Oncologist.

[21] Hasina R., Whipple M.E., Martin L.E., Kuo W.P., Ohno-Machado L., Lingen M.W. (2008). Angiogenic heterogeneity in head and neck squamous cell carcinoma: Biological and therapeutic implications. Lab. Investig..

[22] Rustum Y.M., Tóth K., Seshadri M., Sen A., Durrani F.A., Stott E., Morrison C.D., Cao S., Bhattacharya A. (2010). Architectural heterogeneity in tumors caused by differentiation alters intratumoral drug distribution and affects therapeutic synergy of antiangiogenic organoselenium compound. J. Oncol..

[23] Baruah P., Lee M., Wilson P.O., Odutoye T., Williamson P., Hyde N., Kaski J.C., Dumitriu I.E. (2015). Impact of p16 status on pro- and anti-angiogenesis factors in head and neck cancers. Br. J. Cancer.

[24] Troy J.D., Weissfeld J.L., Youk A.O., Thomas S., Wang L., Grandis J.R. (2013). Expression of EGFR, VEGF, and NOTCH1 suggest differences in tumor angiogenesis in HPV-positive and HPV-negative head and neck squamous cell carcinoma. Head Neck Pathol..

[25] Hauff S.J., Raju S.C., Orosco R.K., Gross A.M., Diaz-Perez J.A., Savariar E., Nashi N., Hasselman J., Whitney M., Myers J.N. (2014). Matrix-metalloproteinases in head and neck carcinoma-cancer genome atlas analysis and fluorescence imaging in mice. Otolaryngol. Head Neck Surg..

[26] Benson E., Li R., Eisele D., Fakhry C. (2014). The clinical impact of HPV tumor status upon head and neck squamous cell carcinomas. Oral Oncol..

[27] Fakhry C., Gillison M.L. (2006). Clinical implications of human papillomavirus in head and neck cancers. J. Clin. Oncol..

[28] Helfrich I., Scheffrahn I., Bartling S., Weis J., von Felbert V., Middleton M., Kato M., Ergün S., Augustin H.G., Schadendorf D. (2010). Resistance to antiangiogenic therapy is directed by vascular phenotype, vessel stabilization, and maturation in malignant melanoma. J. Exp. Med..

[29] Lassen P., Eriksen J.G., Hamilton-Dutoit S., Tramm T., Alsner J., Overgaard J., Danish Head and Neck Cancer Group (DAHANCA) (2010). HPV-associated p16-expression and response to hypoxic modification of radiotherapy in head and neck cancer. Radiother. Oncol..

[30] Tolaney S.M., Boucher Y., Duda D.G., Martin J.D., Seano G., Ancukiewicz M., Barry W.T., Goel S., Lahdenrata J., Isakoff S.J. (2015). Role of vascular density and normalization in response to neoadjuvant bevacizumab and chemotherapy in breast cancer patients. Proc. Natl. Acad. Sci. USA.

[31] Kamat C.D., Green D.E., Warnke L., Thorpe J.E., Ceriello A., Ihnat M.A. (2007). Mutant p53 facilitates pro-angiogenic, hyperproliferative phenotype in response to chronic relative hypoxia. Cancer Lett..

[32] Said R., Hong D.S., Warneke C.L., Lee J.J., Wheler J.J., Janku F., Naing A., Falchook G.S., Fu S., Piha-Paul S. (2013). P53 mutations in advanced cancers: Clinical characteristics, outcomes, and correlation between progression-free survival and bevacizumab-containing therapy. Oncotarget.

[33] Seshadri M., Mazurchuk R., Spernyak J.A., Bhattacharya A., Rustum Y.M., Bellnier D.A. (2006). Activity of the vascular-disrupting agent 5,6-dimethylxanthenone-4-acetic acid against human head and neck carcinoma xenografts. Neoplasia.

