# Supplementary material for: Profiling the Stromal and Vascular Heterogeneity in Patient-derived Xenograft Models of Head and Neck Cancer: Impact on Therapeutic Response
Source: Cancers (Basel). 2019 Jul 6;11(7):951. doi: 10.3390/cancers11070951 (PMC6679003; doi:10.3390/cancers11070951)
Supplement: Supplementary file 1 [file cancers-11-00951-s001.pdf]

cancers

MDPI

Article

Profiling the Stromal and Vascular Heterogeneity in Patient-derived Xenograft Models of Head and Neck Cancer: Impact on Therapeutic Response

Margaret Folaron, Mihai Merzianu, Umamaheswar Duvvuri, Robert L. Ferris, and Mukund Seshadri

Supplementary Files

Methods

HPV amplification and detection by PCR and gel electrophoresis

HPV 16 status was verified in patient samples and matching PDX by PCR. Briefly, DNA was extracted from frozen tissue samples and 10 ng was added to a PCR master mix containing forward (5′-ATTAGTGAGTATAGACATTA-3′) and reverse (5′-GGCTTTTGACAGTTAATACA-3′) primers targeting the E6 region of HPV 16. The PCR reaction was performed as follows: (i) initial denaturation for 7 minutes at 95º C followed by (ii) 40 cycles of 45 second denaturation period at 95º C, 1 minute annealing period at 52º C, 1 minute extension period at 72º C, and (iii) a final extension period for 5 minutes at 72º C. Samples were then resolved by electrophoresis in a 3% agarose gel containing 1μg/mL ethidium bromide. A plasmid containing the HPV-16 E6 region was used as a positive control, while a mock reaction using water was used as a negative control.

Echocardiography

Cardiac imaging was performed with the Vevo® LAZR (VisualSonics Inc., Toronto, ON, Canada) system with a 55 MHz ultrasound (US) transducer. Mice were anaesthetized with 2% isoflurane and secured to the heated imaging platform. The animal's fore and hind limbs were taped to the echocardiography (ECG) leads to acquire the heart rate of the animal. Cardiac imaging mode was activated and long/short axis images were acquired for 100 frames. Following acquisition of datasets, the Vevo® 2100 processing suite was used to perform a Simpson measurement of the heart. Parameters of cardiac function [cardiac output (CO), fractional shortening (FS), ejection fraction (EF)] were calculated by the image processing software (VisualSonics Inc., Toronto, ON, Canada).

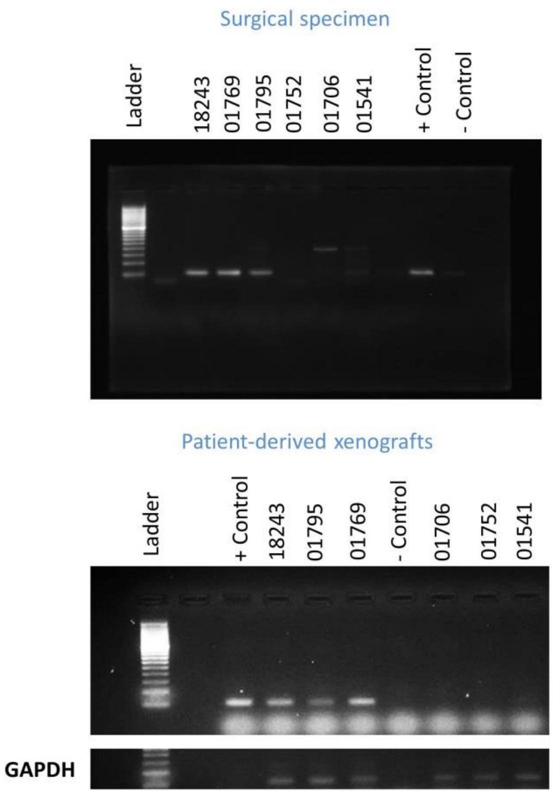

Figure S1. PDX models of HNSCC retain the HPV/p16 status of the original patient tumor. PCR based detection of HPV 16 E6 region in HNSCC surgical specimens and matching six PDX models evaluated in the study. The HPV status of the donor human tumor tissue was retained in the PDX models. In agreement with p16 immunohistochemistry, the three p16+ PDX and their original patient tumors exhibited bands for p16E6.

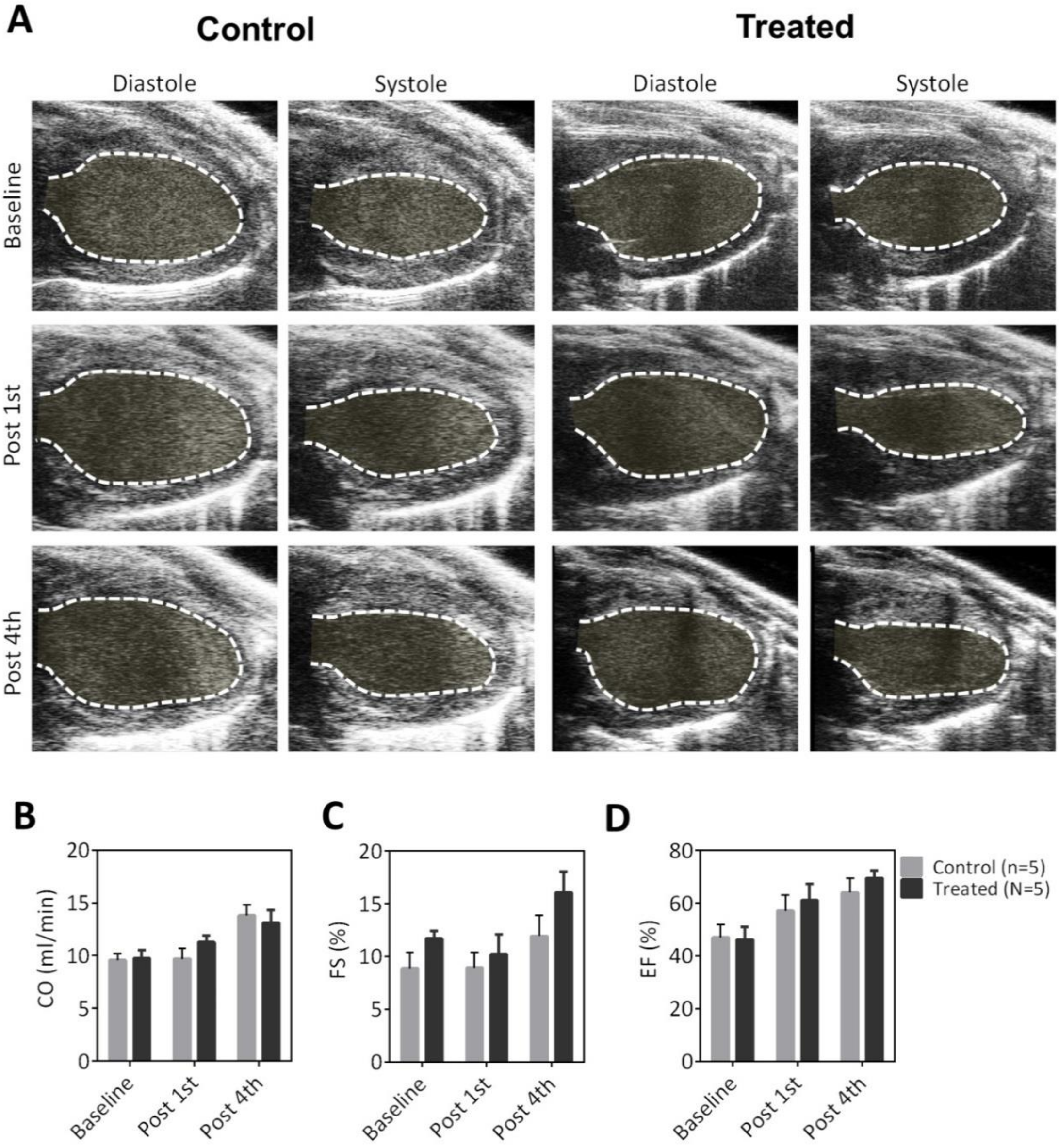

Figure S2. Effect of EPC2407 treatment on cardiovascular function in mice. (**A**) Long axis b-mode ultrasound images of a control (left) and treated (right) mouse heart at baseline (top), 24h following one dose of EPC2407 (middle; 24h post 1st), and 24 h following four doses of EPC2407 (bottom; 24h post 4th). (**B-D**) Corresponding bar graphs of cardiac output (left), fractional shortening (middle), and ejection fraction (right) (n = 5 mice/cohort) are shown in the bottom.

Table S1. Patient characteristics of the PDX models generated in the study.

Patient Info.	01541	01706	01752	18243	01795	01769
Age at Dx	63	67	76	67	51	78
Gender	M	M	M	M	F	M
Primary site	FOM	Parotid	s.c.neck/chest (primary glottis)	Tonsil	Nasal cavity	Supraglottis
Clinical stage	IVA	IVA		IVC	III	III
Treatment	None	None	Radiation	Chemo	None	Chemo

Table S2. Comparative assessment of histology and p16 status of surgical donor tumor tissue and corresponding patient-derived xenografts. We did not observe any relationship between tumor differentiation, vascular phenotype and growth rate. (Well diff – well differentiated SCC; Poorly diff – poorly differentiated SCC; Mod. diff – Moderately differentiated SCC).

	**01541**	**01706**	**01752**	**18243**	**01795**	**01769**
Histology						
Pt. sample	Well diff.	Poorly diff.	Poorly diff.	Mod.diff.	Poorly diff.	Poorly diff.
Estd. PDX	Well diff.	Poorly diff.	Poorly diff.	Mod.diff.	Poorly diff.	Poorly/mod.
HPV status						
Pt. sample	p16-HPV16-	p16-HPV16-	p16-HPV16-	p16+HPV16+	p16+HPV16+	p16+HPV16+
Estd. PDX	p16-HPV16-	p16-HPV16-	p16-HPV16-	p16+HPV16+	p16+HPV16+	p16+HPV16+

Table S3. Patient characteristics of the tissue microarray.

**ID**	**Patient characteristics**	**Site**	**p16**
161	66 yr. old male, 187 mo. survival	Tongue	-
162	47 yr. old male, 89 mo. survival	Gingiva	-
163	63 yr. old female, 13 mo. survival	Oral cavity	-
164	63 yr. old male, 62 mo. survival	Glottis	-
165	49 yr. old male, 78 mo. survival	Tonsil	-
166	68 yr. old male, 177 mo. survival	Base of tongue	+
167	80 yr. old male, 20 mo. survival	Unknown primary	-
168	68 yr. old male, died of other cause	Glottis	-
169	47 yr. old male, 16 mo. survival	Hard palate	-
171	61 yr. old male, 134 mo. alive	Unknown primary	-
172	47 yr. old female, 84 mo. alive	Tonsil	+
18243	67 yr. old male, 70 mo. survival	Tonsil	+
01541	63 yr. old male, 5 mo. alive	Floor of mouth	-
01795	51 yr. old female, 4 mo. alive	Nasal Cavity	+
01706	67 yr. old male, 6 mo. alive	Parotid	-
01769	78 yr. old male, 41 mo. survival	Supraglottis	+
01752	76 yr. old male, 49 mo. survival	Glottis	-

© 2019 by the authors. Licensee MDPI, Basel, Switzerland. This article is an open access article distributed under the terms and conditions of the Creative Commons Attribution (CC BY) license (http://creativecommons.org/licenses/by/4.0/).
